# Association of immune-related adverse events induced by nivolumab with a battery of autoantibodies

**DOI:** 10.1080/07853890.2021.1931956

**Published:** 2021-06-01

**Authors:** Iñigo Les, Mireia Martínez, Alicia Narro, Inés Pérez, Cristina Sánchez, Laura Puntí, Pilar Anaut, Saioa Eguiluz, Alberto Herrera, Severina Domínguez

**Affiliations:** a Department of Internal Medicine, Osakidetza Basque Health Service, Araba University Hospital, Vitoria-Gasteiz, Spain; b Department of Medical Oncology, Osakidetza Basque Health Service, Araba University Hospital, Vitoria-Gasteiz, Spain; c Digestive Cancer Research Group, Bioaraba Health Research Institute, Vitoria-Gasteiz, Spain; d Breast Cancer Research Group, Bioaraba Health Research Institute, Vitoria-Gasteiz, Spain; e Department of Immunology, Osakidetza Basque Health Service, Araba University Hospital, Vitoria-Gasteiz, Spain

**Keywords:** Immune-related adverse events, autoantibodies, nivolumab, immune checkpoint inhibitors, immunotherapy, cancer, prognosis

## Abstract

**Background:**

The aim of this study was to assess the diagnostic performance of an autoantibody battery in patients receiving immune checkpoint inhibitors who experienced immune-related adverse events (irAEs).

**Methods:**

We retrospectively analyzed several variables potentially related to irAEs, namely, demographic, clinical, and laboratory characteristics, including an autoantibody battery (antinuclear, anti-neutrophil cytoplasmic, anti-thyroid antibodies and rheumatoid factor).

**Results:**

Sixty-nine patients (48 men; 61.8 ± 10.9 years at baseline) diagnosed with stage-4 solid-organ cancer and treated with nivolumab were followed up for 12 ± 10.3 months. Thirty-two irAEs were detected in 26 patients (37.5%). Adverse events occurred more commonly in women (62% vs. 27%, *p* = .006), and younger patients (irAEs: 58.1 ± 9.8, no irAEs: 64.1 ± 10.9 years, *p* = .024). Autoantibody battery results were available for 26 patients and were more frequently positive in patients with irAEs (87% vs. 30%, *p* = .009). The positive predictive value, negative predictive value, and diagnostic accuracy of the battery were 82.3%, 77.8%, and 80.8%, respectively. Among the 64 patients with an evaluable response, 23 (38.5%) experienced tumour progression, this being less frequent in patients with irAEs (19% vs. 48.5%, *p* = .03). Overall survival was higher in patients developing irAEs (HR = 1.88, *p* = .05).

**Conclusion:**

Positivity in a readily available autoantibody battery may be associated with the occurrence of irAEs.

**KEY MESSAGES**
- Positivity in an accessible and inexpensive autoantibody battery including antinuclear, anti-neutrophil cytoplasmic, anti-thyroid antibodies and rheumatoid factor may be associated with the occurrence of immune-related adverse events.
- Patients with cancer on immune checkpoint inhibitors experiencing immune-related adverse events showed a lower risk of progression and better overall survival than patients not experiencing this type of adverse effect.

## 1. Introduction

### 1.1. Background

Immune checkpoint inhibitors (ICIs) are a family of drugs increasingly widely used in the treatment of solid-organ cancer [1]. Their mechanism of action consists of the blockade of inhibitory membrane receptors or checkpoints, such as cytotoxic T-lymphocyte antigen 4 (CTLA-4) and programmed cell death protein (PD-1), which act physiologically as negative regulators of the cytotoxic lymphocytes in charge of controlling malignant cells [2]. By blocking these inhibitory proteins and their ligands, such as programmed death-ligand 1, the effect of ICIs has been compared to “releasing the brake” on the immune system [3]. As a result, ICIs trigger an enhanced response with intended antitumoral consequences but, at the same time, may induce a myriad of immunological toxic effects known as immune-related adverse events (irAEs) [4].

To date, most studies on ICIs have focussed on clinical efficacy [5]. Concerning toxicity, though many patients receiving ICIs experience some form of irAEs, which hinder or even prevent ICI administration, predictors of irAEs are not well established [6]. On the other hand, patients diagnosed with autoimmune diseases, some of which are *per se* risk factors for malignancy [7], have been systematically excluded from the pivotal clinical trials relating to ICIs [8]. Despite this hampering access to ICIs, the available evidence indicates that survival is better in patients who experience irAEs than those who do not [9]. Unfortunately, immunosuppressant therapy prescribed for managing irAEs may decrease the antitumoral effect of ICIs due to an immune-system damping mechanism [10]. Hence, there is a need for more accurate clinical and laboratory characterization of patients with cancer at risk of developing irAEs.

The potential role of autoantibodies as diagnostic biomarkers of toxicity has been separately reported for each form of irAE. Specifically, immune-mediated thyroiditis has been related to the presence of anti-thyroid antibodies (ATA) before ICI initiation [11]. Similarly, the PD-1 pathway blockade has been shown to promote the expansion of the humoral response leading to increases in autoantibody titres and clinical activity in some autoimmune diseases [12]. Levels of generic autoantibodies, such as antinuclear antibodies (ANA), are elevated in autoimmune diseases and occasionally cancer [13] and may serve as an overall indicator of autoimmune status. In patients with melanoma, a tumour with marked immunogenicity, ANA and ATA positivity has been associated with a favourable response to interferon [14]. Therefore, we hypothesized that the presence of generic autoantibodies may predict the risk of developing irAEs.

### 1.2. Objectives

This study aimed to assess the diagnostic performance of a battery of autoantibodies including ANA, anti-neutrophil cytoplasmic antibodies (ANCA), rheumatoid factor (RF) and ATA in patients with cancer receiving ICIs who experienced irAEs. We also sought to compare the cancer-related prognosis of patients who did and did not experience irAEs.

## 2. Materials and methods

### 2.1. Study design, setting and participants

The current study corresponds to the retrospective single-centre pilot phase of a prospective multicenter project registered on clinicaltrials.gov (name: *AUTENTIC;* identifier*:* NCT03868046). The study protocol was designed between the departments of Internal Medicine and Medical Oncology at Araba University Hospital, a tertiary care hospital, and approved by the Ethics Committee of each participating centre and the Spanish Agency of Medicines and Medical Devices (reference number: ILB-NIV-2018-01). The data that support the findings of this study are openly available in “Mendeley data” at http://dx.doi.org/10.17632/46vj8r9dkx.1.

Between August 2015 and March 2018, eligible patients were selected from the oncology outpatient clinic of Araba University Hospital, where they had been referred from secondary hospitals in the area and other departments within the hospital. As inclusion criteria, patients were required to have been diagnosed with solid-organ cancer amenable to treatment with an ICI as per standard practice and sign an informed consent form. Exclusion criteria were an estimated baseline survival of fewer than 3 months, an absolute contraindication to receiving ICIs (i.e. known immediate hypersensitivity, an active autoimmune disease with potentially life-threatening involvement), and ongoing immunosuppressant or glucocorticoid therapy (i.e. dexamethasone at a dose higher than 1.5 mg/day or equivalent).

### 2.2. Endpoints and variables

The primary endpoint of the study was the proportion of patients who developed irAEs after receiving at least one dose of ICI and to identify predictive factors of irAEs. The secondary endpoint of the study was the response to ICIs according to immune-Response Evaluation Criteria in Solid Tumours (iRECIST) criteria and overall survival depending on whether or not patients developed irAEs.

Based on previous evidence, several variables potentially related to irAEs such as demographic characteristics (sex, age), clinical data (cancer type and stage, prior autoimmune diseases), and baseline laboratory parameters (complete blood cell count, glomerular filtration rate, and bilirubin and thyroid hormone levels), measured at a maximum of 1 week before ICI initiation, were incorporated into the statistical analysis. Among patients from whom blood samples were available for analysis, the laboratory variables included results of a battery of autoantibodies: ANA, ANCA, RF and ATA (namely, anti-thyroid-peroxidase and anti-thyroglobulin antibodies).

We defined irAEs in accordance with the Common Terminology Criteria for Adverse Events version 5.0, in which grades 1 and 2 are categorized as low-grade adverse events and grades 3 and 4 as high-grade adverse events. The treatment response was defined as the overall response rate in accordance with the iRECIST version 1.1 [15].

### 2.3. Follow-up protocol

After the initial assessment visit, all patients included were monitored regularly, with appointments scheduled depending on the dosing interval and the appearance of toxicity or other complications, at the discretion of the attending physician. During follow-up, measurement of the autoantibodies did not follow a predefined schedule; rather, when a patient experienced an irAE, attending physicians were encouraged to request an autoantibody battery and, additionally, researchers made efforts to obtain a recent blood sample to measure the autoantibodies included in the battery. In all cases in which we ran the battery, regardless of whether the patient had developed irAEs or not, the samples had been taken after the first dose of ICI and not at baseline.

### 2.4. Techniques for autoantibody measurements

In agreement with international recommendations [16], detection of ANA was based on indirect immunofluorescence (IFI) assays performed on a fully automated system (EUROPattern, Euroimmun^®^). The patient’s serum was incubated with a HEp-2 cell line as substrate attached to a slide at a screening dilution of 1:160. After washing, a fluorescein-conjugated anti-human IgG was added; this bound to antibodies from the patient’s serum that had previously reacted with the substrate antigens on the slide. Next, the slide was examined under an ultraviolet microscope on a viewer, and resulting images were digitally recorded with the aid of a middleware (EUROLabOffice, Euroimmun^®^). If fluorescence was detected in one or more screening dilutions, the serum was serially diluted and re-analyzed until less than half of the cells on the slide showed detectable fluorescence. The ANA titre was then reported as the dilution before this endpoint.

Similarly, ANCA were screened by IFI in the aforementioned fully automated system (EUROPattern) incubating the patient’s serum at different dilutions on a slide in combination with neutrophils fixed with ethanol, formalin, and HEp-2 cells as substrate. The cut-off for ANCA positivity was set at a titre of 1:20. Using the Phadia 250 analyzer (Thermo Fisher^®^), positive and doubtful results were confirmed or excluded by an immunoassay test for antibodies to myeloperoxidase and proteinase 3, depending on IFI patterns.

Levels of RF were measured in serum samples using an automated rheumatoid factor test (ARCHITECT system, Abbott^®^). This technique consisted of agglutination immunoturbidimetry involving an antigen-antibody reaction between the RF present in the sample and the human IgG isotype denatured and adsorbed on latex particles. The resulting agglutination was detected as a change in absorbance (572 nm), which was proportional to the amount of RF in the serum. The concentration was then obtained by interpolation on a calibration curve obtained from a reference sample. The result was considered positive if the RF value was greater than 30 IU/ml.

The ATA testing involved measuring two types of autoantibodies, namely, anti-thyroid peroxidase and anti-thyroglobulin antibodies. Anti-thyroid peroxidase antibodies were measured using a chemiluminescent microparticle immunoassay with an acridinium-labeled human IgG conjugate in the Abbott^®^ Alinity Diagnostic System. The result was expressed as relative light units, which are proportional to the concentration of antibodies in the sample, and the upper limit of the reference range was 5.65 IU/ml. For the anti-thyroglobulin antibodies, we used an electrochemiluminescence immunoassay with ruthenium as a marker in the Roche^®^ Cobas 6000 Analyser, with a reference range between 0 and 115 IU/ml.

### 2.5. Statistical analysis

Quantitative variables were reported as mean ± standard deviation or median (interquartile range) and compared between groups using Student’s *t*-test for unpaired data or the Mann–Whitney *U* test depending on whether data were normally distributed. Correlations between quantitative variables were assessed using Spearman’s *Rho*. Categorical variables were expressed as frequencies (percentages) and compared between groups using the *χ*^2^-Pearson’s and Fisher’s exact tests depending on the expected cell frequencies. Covariates reaching statistical significance in the bivariate analysis were included in a multivariate logistic regression that was restricted to a subject-to-variable ratio of 20:1. The diagnostic accuracy of the model to predict irAEs was assessed using sensitivity, specificity, area under the receiver operating characteristic (ROC) curve, predictive values, and likelihood ratios.

Overall survival was assessed using Kaplan–Meier analysis and compared between patients with and without irAEs using the log-rank Mantel–Cox test. Two-sided hypothesis tests were performed, and the significance level was set at 5%. The statistical analysis was conducted using IBM SPSS Statistics version 25.0 (Chicago, IL).

## 3. Results

### 3.1. Descriptive data

Table 1 summarises the general characteristics of the patients in our cohort. Briefly, 69 patients (overall baseline mean age of 61.8 ± 10.9 years; 48 men [69.5%]), all of them diagnosed with stage-4 cancer and treated with the anti-PD-1 agent nivolumab (3 mg/kg every 2 weeks), were enrolled and followed up for 12 ± 10.3 months from the time of ICI initiation and 16.4 (10.6–33.2) months from the time of diagnosis. Before starting on nivolumab, seven patients (10.1%) had an inactive autoimmune disease, which did not contraindicate the therapy. The primary cancer was non-small cell lung cancer in 47 (68.1%), melanoma in 11 (15.9%), renal cell carcinoma in 9 (13%), and head and neck cancer in 2 (2.8%) cases.

**Table 1.** Baseline characteristics of the patients included in the study (*n* = 69)^a^.

Demographic features	
Age, years	61.8 ± 10.9
Female/male	21 (30.4)/48 (69.6)
Previous inactive autoimmune disease^b^	7 (10.1)
Laboratory parameters	
Lymphocyte count, /mm^3^	1728 ± 814
Eosinophil count, /mm^3^	160 ± 147
Cancer-related features	
Stage 4	69 (100)
Follow-up after cancer diagnosis, months	16.4 (10.6–33.2)
Follow-up after ICI initiation, months	12 ± 10.3
Treatment with nivolumab	69 (100)
Type of cancer	
Non-small cell lung cancer	47 (68.1)
Melanoma	11 (15.9)
Renal cell carcinoma	9 (13)
Head and neck cancer	2 (2.8)

Quantitative variables are expressed as mean ± standard deviation or median (interquartile range) depending on whether the data were normally distributed; categorical variables are expressed as frequencies (percentage).

^a^Baseline characteristics of the 26 patients for whom autoantibody battery results were available were similar to those of the whole cohort.

^b^Includes 2 cases of rheumatoid arthritis, 2 of autoimmune thyroiditis, 1 of polymyalgia rheumatica, 1 of immune-based glomerulonephritis and 1 of polymyalgia rheumatica concurrent with IgA nephropathy.

ICI: immune checkpoint inhibitor.

### 3.2. Incidence of irAEs

During the follow-up, we detected 32 cases of irAEs in 26 patients (37.6%). Six patients experienced at least two different irAE episodes. The irAEs appeared at a median of 9.1 (3–25.8) weeks after the start of treatment and patients had received a median of 22 (7–26) ICI cycles before the performance of the autoantibody battery. In patients who developed irAEs, the autoantibody battery was performed as a median of 38.3 (9.7–46.9) days after identification of the irAE. Differences in the number of nivolumab cycles between patients with and without irAEs did not reach significance.

All the irAEs identified were organ-specific, corresponding to 8 cases of thyroiditis (25%), 6 of enterocolitis (18.8%), 5 of inflammatory arthritis (15.6%), 4 cases each of dermatitis and pneumonitis (12.5% each), 2 cases each of nephritis and hepatitis (6.25% each), and 1 of autoimmune thrombocytopenia (3.1%). Most of the irAEs detected were low- rather than high-grade adverse events (26/32 [81%] vs. 6/32 [19%], respectively).

### 3.3. Autoantibody positivity and other factors associated with irAEs

In the bivariate analysis (Table 2), the occurrence of irAEs was associated with female sex (13/21 [61.9%] vs. 13/48 [27.1%], *p* = .006) and younger age at the time of starting nivolumab therapy (58.1 ± 9.8 years in patients with irAEs vs. 64.1 ± 10.9 years in patients without irAEs, *p* = .024). In contrast, patients with an inactive autoimmune disease before nivolumab initiation were not more likely to develop irAEs (*p* = .766). Further, irAEs were also not related to type of cancer (*p* = .445), baseline lymphocyte count (*p* = .364) or baseline eosinophil count (*p* = .993).

**Table 2.** Results of the bivariate and multivariate analyses of factors associated with immune-related adverse events (irAEs).

Bivariate analysis			
Variable	OR	95%CI	*p*-Value
Female sex^a^	4.37	1.47–12.97	.008
Age at ICI initiation^a^	0.95	0.90–0.99	.033
Previous inactive autoimmune disease	1.27	0.26–6.19	.766
Lymphocyte count	1.00	0.99–1.00	.364
Eosinophil count	1.00	0.99–1.01	.993
Type of cancer	1.10	0.77–1.57	.445
Autoantibody positivity^b^	16.33	2.19–121.42	.006
			
Multivariate analysis^b^			
Female sex	4.98	0.36–69.52	.232
Age at ICI initiation	0.93	0.82–1.04	.211
Autoantibody positivity	46.61	2.48–876.10	.010

^a^The bivariate analysis for sex and age performed in the subgroup of 26 patients for whom autoantibody battery results were available showed a trend towards a higher risk of developing irAEs in women (OR 5.14, 95%CI 0.82–32.30, *p* = .081) but not in younger patients (OR 0.97, 95%CI 0.89–1.05, *p* = .461).

^b^Analysis performed with the 26 patients for whom the autoantibody battery results were available.

OR: odds ratio; CI: confidence interval; ICI: immune checkpoint inhibitor.

**Figure 1.**
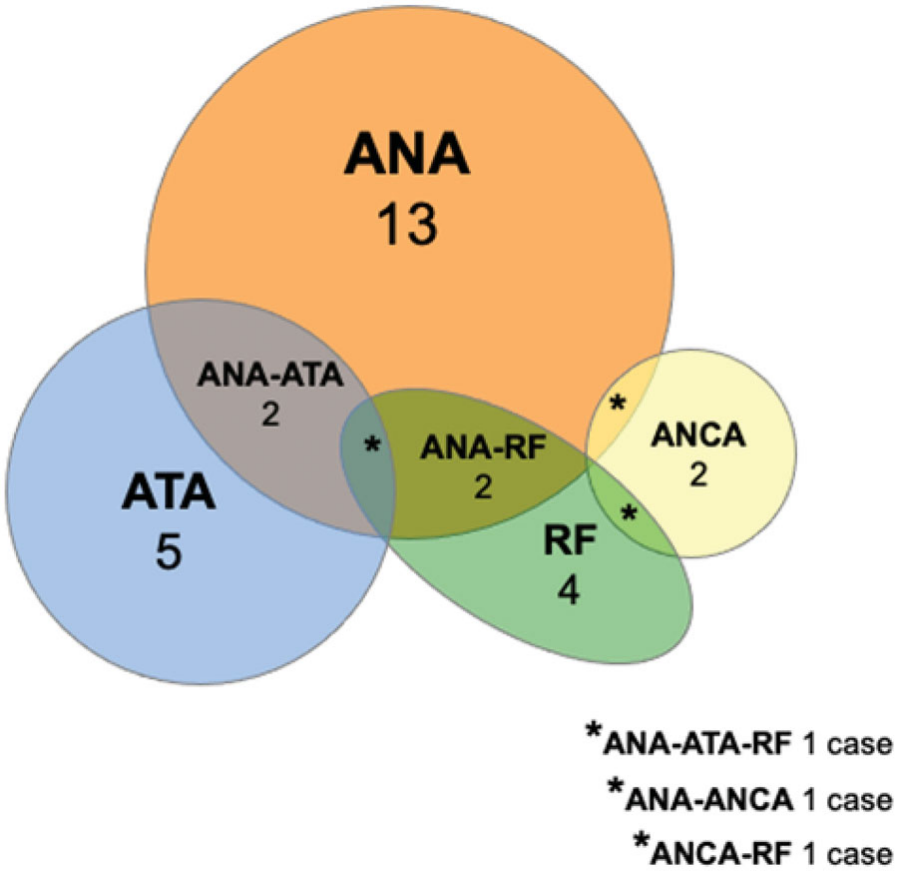
Prevalence of the autoantibodies detected in the 17 patients with autoantibody positivity in the battery used: 13 (76.4%) cases with ANA, 5 (29.4%) cases with ATA, 4 (23.5%) cases with RF, and 2 (11.7%) cases with ANCA. There were 2 (11.7%) cases each of overlap between ANA and ATA and between ANA and RF, and 1 (5.8%) case each of overlap among ANA, ATA and RF, between ANA and ANCA and between ANCA and RF. Out of the 17 patients with autoantibody positivity in the battery, 15 were found to be positive after ICI initiation, and 2 cases were known to be already positive at baseline, the positivity being confirmed during the follow-up. ANA: antinuclear antibodies; ATA: anti-thyroid antibodies; RF: rheumatoid factor; ANCA: anti-neutrophil cytoplasmic antibodies.

Autoantibody battery results were available for only 26 patients (16 of them with irAEs and 10 without irAEs), this low number being attributable to the retrospective nature of the study. Baseline characteristics of these 26 patients were similar to those of the whole cohort (mean age of 59.2 ± 10.5 years, 15 men [57.7%], 5 of the 26 patients [19.2%] diagnosed with a previous inactive autoimmune disease). The autoantibody battery results were more frequently positive in patients with irAEs than in those without irAEs (respectively, 14/16 [87.5%] vs. 3/10 [30%] of patients being positive for at least one of the antibodies studied, *p* = .009). This positivity corresponded to 13 cases with ANA, 5 cases with ATA, 4 cases with RF, and 2 cases with ANCA. Antigens related to ANA positivity were double-stranded DNA in 7 cases, Ro in 5 cases and overlap among topoisomerase I, U3 RNP/fibrillarin and RNA polymerase I in 1 case. The two patients with positive ANCA expressed anti-myeloperoxidase antibodies in the immunoassay test and neither of them developed an irAE. There was a marked overlap among the autoantibodies included in the battery (Figure 1).

The frequency of positivity for autoantibodies was similar in patients with high- and low-grade irAEs (4/6 [66.7%] vs. 17/26 [65.4%], *p* = .952). Moreover, there was no correlation between the irAE grade and the titre of ANA measured by IFI (correlation coefficient, *Rho* = −0.332, *p* = .605).

The multivariate analysis applied to the subgroup of 26 patients with data available on autoantibodies identified the autoantibody battery results as the only independent predictor of irAEs (Table 2).

### 3.4. Diagnostic performance of the autoantibody battery

The battery of autoantibodies, assessed in all cases after the first dose of nivolumab, showed a sensitivity of 87.5%, specificity of 70%, positive predictive value of 82.4%, negative predictive value of 77.8%, positive likelihood ratio of 2.92, negative likelihood ratio of 0.18, and diagnostic accuracy of 80.8%.

**Figure 2.**
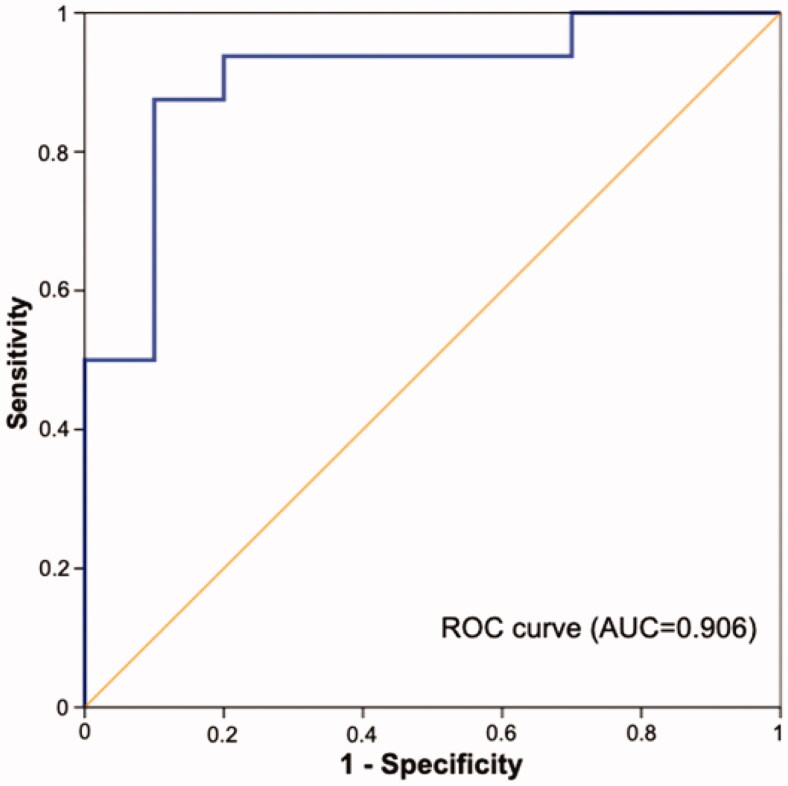
Area under the receiver operating characteristic curve for the predictive model of immune-related adverse events including age, sex and autoantibody battery results. ROC: receiver operating characteristic; AUC: area under the curve.

In the predictive model based on the three factors associated with irAEs in the bivariate analysis (namely, age, sex and the autoantibody battery), the area under the ROC curve was 0.906 (Figure 2).

### 3.5. Cancer-related outcomes

**Figure 3.**
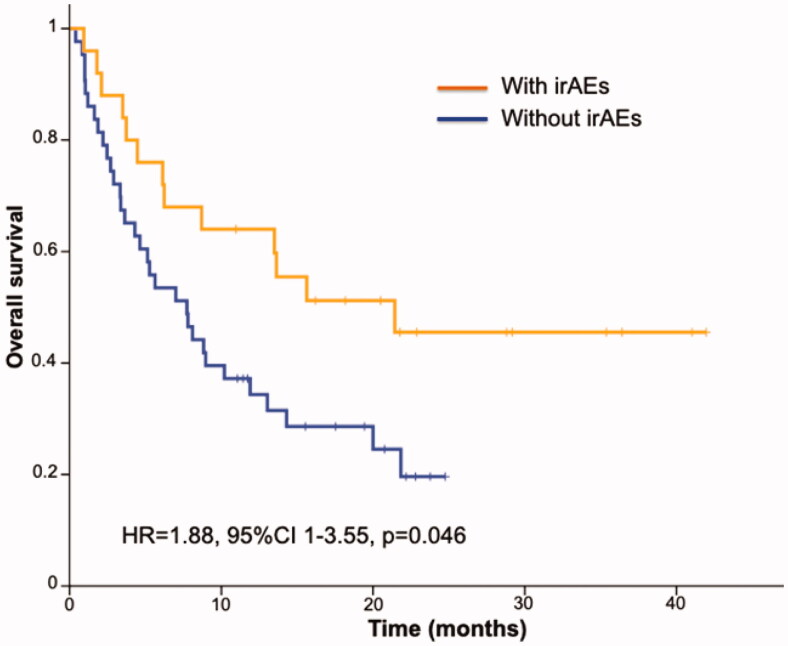
Kaplan–Meier estimated overall survival curves comparing patients with and without immune-related adverse events. irAEs: immune-related adverse events; HR: hazard ratio; CI: confidence interval.

Regarding treatment response, we observed partial response in 30 (43.5%), complete response in 2 (2.9%), stable disease in 9 (13%), and progressive disease in 23 (33.3%) patients. In accordance with iRECIST criteria, the response was not evaluable in 5 (7.2%) cases, and these were excluded from the analysis. In the rest (*n* = 64), the risk of progression was significantly higher in patients without irAEs (19/41 [46.3%] vs. 4/23 [17.4%] in patients with irAEs, *p* = .03). Likewise, patients with irAEs showed greater overall survival (HR = 1.88, 95%CI 1–3.55, *p* = .05 [Figure 3]). Positivity for autoantibodies did not, however, predict a better prognosis in terms of progression-free or overall survival (data not shown).

## 4. Discussion

The main finding of this study is that the positivity for any of the autoantibodies included in the battery used, namely, ANA, ANCA, RF and ATA, may be associated with irAEs in patients receiving nivolumab. This autoantibody battery, combined with other potential predictors of toxicity, showed an acceptable diagnostic accuracy of almost 81% and an area under the ROC curve of over 0.9.

Our bivariate analysis identified three risk factors for the development of irAEs: female sex, younger age and autoantibody positivity. Regarding sex, previous studies have shown that women are more likely to experience irAEs induced by CTLA-4 and PD-1 inhibitors [17]. This susceptibility is similar to other autoimmune conditions in the general population [18] and maybe explained by genetic and hormonal mechanisms [19]. Recently, evidence of a higher incidence of irAEs in premenopausal women has strengthened the view that sex hormones play a role in the pathogenesis of immune-related toxicity [20].

In the case of age, while older patients may exhibit an impaired tumour response [21], the impact of ageing on the risk of irAEs remains unknown. In our cohort, young patients presented with irAEs more frequently than the elderly. This finding could be attributed to a vigorous immune system able to orchestrate a pathologic reaction in young people, as in most autoimmune diseases [22]. In contrast, previous research has suggested that the risk of irAEs may be higher in patients of advanced age [23]. Reduced functional reserve, age-related comorbidities, interactions among multiple drugs and the phenomenon of immune-senescence might predispose elderly patients to develop irAEs [24].

Regarding the utility of the battery, the results of our multivariate analysis agree with other retrospective research showing that autoantibodies are an independent predictive factor for irAEs in patients receiving ICIs [25]. De Moel et al. reported a trend to an association between positivity in a large battery of 23 autoantibodies and irAEs related to ipilimumab in 133 patients with melanoma [26]. In accordance with the thyroiditis model [27], De Moel et al. also found a relationship between ipilimumab-induced ATA and thyroid dysfunction under subsequent anti-PD-1 therapy. These findings were confirmed by Toi et al., who showed that pre-existing positivity for commonly studied autoantibodies (ANA, RF and ATA) was associated with irAEs in a cohort of 137 patients with non-small cell lung cancer treated with nivolumab [28]. Our findings may be of interest because they add to the evidence supporting the clinical application of autoantibodies as a potential biomarker of toxicity to any tumour amenable to ICI therapy. Taking a pragmatic line, we favour using generic autoantibodies, such as those included in our battery, as the first level of screening for irAEs.

Consistent with other available evidence [29], our results support the view that the prognosis of patients with irAEs is better than in those without irAEs. On the other hand, we did not observe the same favourable association between autoantibody positivity and cancer-related outcomes. Unlike the exclusion criteria applied in most clinical trials [8], selected patients with autoimmune features could be suitable candidates for receiving ICIs, especially if we apply tools capable of detecting individuals with a problematic risk-benefit ratio. There is still a need to improve our understanding of the relationship among autoimmunity profile, risk of irAEs and ICI efficacy [30].

Our study has several methodological limitations that should be recognized. First, the observational retrospective design does not allow us to draw conclusions about causality, and the single-centre approach limits generalization of the results to other settings. Second, the lack of an association between the autoantibody battery and cancer-related outcomes may be due to the heterogeneity of our population, with several types of cancer, and a low statistical power arising from the relatively small sample size. Third, the timing of autoantibody measurement did not follow a predefined schedule at different time points, and hence, it varied between patients. Likewise, we were unaware of the autoantibody patients’ status at baseline. Fourth, we were unable to retrieve blood samples to measure the autoantibody battery in a substantial proportion of patients. Finally, our results are based only on patients receiving nivolumab therapy, and therefore may not be generalizable to patients on other ICIs. We hope to overcome these shortcomings in the next prospective multicenter phase of our current research project.

## 5. Conclusions

Positivity in an accessible and inexpensive autoantibody battery composed of ANA, ANCA, RF and ATA may be associated with the occurrence of irAEs. The predictive power of this battery is currently being evaluated in a multicenter prospective study conducted by our group (*AUTENTIC*, NCT03868046). Consistent with previous research, patients on nivolumab with irAEs showed a lower risk of progression and better overall survival than patients not experiencing this type of adverse effect.
